# A question of time: How demographic faultlines and deep-level diversity impact the development of psychological safety in teams

**DOI:** 10.3389/fpsyg.2022.765793

**Published:** 2022-09-29

**Authors:** Rebecca Gerlach, Christine Gockel

**Affiliations:** ^1^Department of Psychology, Chemnitz University of Technology, Chemnitz, Germany; ^2^Berlin School of Management, SRH University Berlin, Berlin, Germany

**Keywords:** psychological safety, development, team member personality, competency, growth curve modeling, demographic faultlines

## Abstract

Psychological safety (PS) is a shared belief among team members that it is safe to take interpersonal risks. It can enhance team learning, experimentation with new ideas, and team performance. Considerable research has examined the positive effects of PS in diverse organizational contexts and is now shifting its focus toward exploring the nature of PS itself. This study aims to enhance our understanding of PS antecedents and development over time. Based on the model of team faultlines and research on team diversity, we examined the effects of demographic faultlines, team member personality, and member competencies on the development of PS. Over 5 months, 61 self-managed teams (*N* = 236) assessed their PS at the beginning, midpoint, and end of a research project. Results of a multilevel growth curve model show that PS decreased from project beginning to end. Initial levels of PS were especially low when teams had strong demographic faultlines and when team members differed in neuroticism. PS decreased more strongly over time when team members were diverse in agreeableness and assessed their task-related competencies to be relatively high. Our study identifies time and team composition attributes as meaningful predictors for the development of PS. We present ideas for future research and offer suggestions for how and when to intervene to help teams strengthen PS throughout their collaboration.

## Introduction

Psychological safety (PS) is a key factor for successful collaboration (Bergmann and Schaeppi, 2016). It is defined as team members’ shared belief that their environment is conducive to interpersonally risky behaviors such as speaking up, asking for help, or owning up to mistakes (Edmondson and Lei, 2014). In teams with a high level of PS, members send and receive signals to each other, prompting them to share ideas or admit mistakes, compared to teams with a low level of PS. They report more errors because they are not afraid of being seen as incompetent or overstrained by other team members or their team leader (Edmondson, 1999). When team members experience PS, they regard the team to be a protected, safe context in which they can take risks without the potential for negative consequences (Edmondson, 1999; Garvin et al., 2008). Thus, PS forms the basis for a supportive learning environment for individuals, teams, and organizations (Edmondson, 1999; Garvin et al., 2008; Frazier et al., 2017; Harvey et al., 2019).

Although considerable theory and research link PS with numerous organizational outcomes, for the most part, extant studies are based on state descriptions at one specific point in time (Frazier et al., 2017). Recently, researchers’ attention has shifted to PS as an emerging phenomenon with limits in its dynamics and mode of action over time (e.g., Gerlach and Gockel, 2018; Deng et al., 2019; Harvey et al., 2019; Higgins et al., 2020). For example, researchers have focused on differences within team members’ perception of PS and showed that belonging to a leader’s in-group (compared to an out-group) protected team members from the negative effect of task conflict on PS (Gerlach and Gockel, 2018). Also, PS had the most positive effect on organizational performance over time when being relatively low (as compared to high) combined with high levels of employees feeling accountable for their actions or decisions (in a 3-year study by Higgins et al., 2020). Moreover, a recent interview study explored how leaders experienced and created PS under extreme situations such as the COVID-19 pandemic allowing PS to unfold its positive impact on learning opportunities together with environmental and organizational factors (Weiner et al., 2021). Altogether, these findings have highlighted the importance of subgroup dynamics and boundary conditions for the creation of PS by using more complex study designs.

Regarding the development of PS, theory suggests that PS—as an emergent state—is bound to time and changes as a function of what happens in the team (see a dynamic model of team learning climate, Harvey et al., 2019). However, empirical evidence is mixed so far. In a study of innovative project teams, no changes in PS were found (Edmondson and Mogelof, 2005). Another study indicates that PS decreases, as it co-evolves with network ties over time (Schulte et al., 2012). Thus, we hope to shed light on these contradictory findings by exploring the following questions: How does PS form and develop in teams? Which antecedents related to team member characteristics facilitate changes in PS?

This study is designed to address these questions using an approach that is grounded in theories on team learning and team diversity: More specifically, we build on the theoretical model of group faultlines (Lau and Murnighan, 2005) that links subgroup dynamics from group diversity (what members bring into the group) to the formation and development of PS. We tested the effects of demographic faultlines, team member personality characteristics, and team member competencies as predictors for the formation and development of PS in teams. In our study, we examined self-managed project teams from their formation until task completion. Employing a multilevel growth curve model, we investigate initial levels and changes in PS over time. This study advances our understanding of how member diversity and time affect the development of PS, with an emphasis on team learning opportunities.

### Time, team development, and psychological safety

The perception of PS by single members, subgroups, and across the entire team forms early after team formation and continues to develop over time (Harvey et al., 2019). Teams might grow together. Yet, critical incidences or transitions might impair a desirable positive development (Marks et al., 2001; Harvey et al., 2019). For example, whereas leaders can facilitate the emergence of PS by inviting members to discuss opposing views (Chen et al., 2011), conflicts or disagreements between team members can reduce the chance for a learning climate within a team (Bunderson and Boumgarden, 2010).

Teams are bound to time and task progress in their work: The beginning, the midpoint, and the end of a project have been identified to be important for team development and the design of team interventions (e.g., Gersick, 1988; Hackman and Wageman, 2005; Bush et al., 2018, for an overview on team development theories, see Delice et al., 2019). As described in the punctuated equilibrium model, teams pay attention to time and organize their work in response to the time that is available to complete their task (Gersick, 1988). Specifically, team members are sensitive to the passage of time and the corresponding increase in time pressure and may address this by changing their strategies or approaches to the task. Time is found to be understood as part of the task that needs to be managed by teams stressing the midpoint as one important turning point for team processes and outcomes (Okhuysen and Waller, 2002; Ford and Sullivan, 2004). As such, teams show a particular tendency to make radical changes around the midpoint, for example in their informal leadership structures, marking a transition between two rather balanced phases of collaboration (Gersick, 1988). With the passage of the midpoint, teams can change their motivational frame from being task-focused to performance-focused, adjusting their exploratory search for information during these phases accordingly (Knight, 2015). Team mood is the driver of this change of focus over time. Teams might ask, around the temporal midpoint, “Did we do a good job so far, and are we satisfied with where we stand? Did we put enough resources into searching for information and ideas on how to solve the task, or do we need more information for a solid decision?,” as compared to “Can we move on to the performance phase?” These aspects might be related to team members’ specific learning behavior *search for information* that is bound to the ongoing performance of the task. In this study, we focus on PS, which is the supportive environment for learning throughout a team project. We examine the kind of change that PS shows during a project and the antecedents for initial levels and changes in PS.

To date, only three studies have investigated the temporal development of PS in project groups: First, innovative teams were examined from the beginning of a project to the end (Edmondson and Mogelof, 2005). In this ground-breaking study, PS was found to be stable across three measurements (*M_T1_* = 3.06; *M_T2_* = 3.07; *M_T3_* = 3.01). Second, the relation between PS and friendship ties in social networks was examined in consulting teams working together for the time of a project (Schulte et al., 2012), with PS decreasing from beginning to the end (*M_T1_* = 3.81; *M_T2_* = 3.59; *M_T3_* = 3.37; no tests of significance reported). A third study found only a moderate correlation (*r* = 0.27) between two PS assessments taken at the individual level 6 weeks apart (Liang et al., 2012), which is an indication that PS assessments have changed.

We believe that PS should diminish in teams from the beginning to the end of a project. Research on the need for closure (Pierro et al., 2003) implies that when teams need to make decisions under time pressure, they tend to cut off discussions and strive for task completion. Therefore, we predict that, as time passes, time pressure will contribute to an atmosphere of closed-mindedness in the group. As a deadline approaches, team members should be less likely to pose questions, speak up, or share controversial opinions and, if they continue to do so, are more likely to be sanctioned by their fellow team members. Supporting this prediction, research also shows that novel contributions are more positively valued when being made early during collaboration compared to past the midpoint when this might disrupt performance and lead to frustration (Ford and Sullivan, 2004). Sharing novel contributions is a learning behavior closely linked to PS in teams. Thus, PS might develop similarly in project teams. Therefore, we propose the following.

*Hypothesis 1*: As time passes in a team project, psychological safety decreases.

### Demographic faultlines predict the formation of psychological safety

Group members are sensitive to signals of PS from the very beginning of their collaboration (Gersick, 1988; Hackman and Wageman, 2005). They assess each other instantly depending on overt demographic attributes such as gender, ethnicity, age, tenure, and—if available—functional background (Lau and Murnighan, 2005). These overt demographic characteristics are summarized under the term surface-level diversity or demographic diversity, which can be easily assessed after a brief contact (Bell, 2007). Specifically, team members use demographic differences and overt skills to classify themselves and others into homogeneous (sub-)groups with which they can identify. Research shows that demographic diversity may result in improved decision-making and problem-solving (Watson et al., 1993), but it is also associated with reduced interpersonal liking, intergroup communication, and team cohesion (Tsui and O’Reilly, 1989; Tsui et al., 1992; Smith et al., 1994), as well as an increase in team conflict (Jehn, 1995).

For a long time, different conceptualizations and measurements have led to an enormous body of inconsistent findings integrated for the first time by the theory and unified measurement of team faultlines (Thatcher and Patel, 2012). The term “faultline” comes from the field of geography and refers to the boundary zones between tectonic plates of continents. Transferred to the context of group research, it describes a hypothetical line that potentially splits a team into homogeneous subgroups according to team members’ similarity along with a set of multiple attributes (Lau and Murnighan, 1998). The term “team faultlines” refers only to the joint effect of surface-level attributes such as gender, age, ethnicity, or functional background that serve as the basis for the categorization processes of team members into possible homogeneous subgroups (Lau and Murnighan, 1998, 2005). To be more specific about the type of faultlines, Lau and Murnighan (1998) introduced the term demographic faultlines which can be weak or strong. As such, demographic faultline strength was found to have a consistently negative effect on team processes and outcomes (Thatcher and Patel, 2012).

The model of demographic faultlines (Lau and Murnighan, 2005) links team diversity with perceptions of PS on a theoretical level. The model proposes that demographic faultlines negatively affect PS as well as team learning, anticipated team performance, and team satisfaction (Lau and Murnighan, 2005). In teams with a strong demographic faultline, team members identify, communicate, and share information more often with members of the same subgroup than with members from other subgroups (Lau and Murnighan, 1998, 2005), which should undermine the overall team perception of PS. The model of group faultlines suggests further that demographic faultlines affect PS at the start of a team project, because it is essential for members to find productive subgroups right away, and they often do so based on a preliminary assessment of overt surface-level attributes (Lau and Murnighan, 1998, 2005). Thus, we propose the following.

*Hypothesis 2*: Demographic faultline strength is negatively associated with initial levels of psychological safety.

### Team member personalities and competencies predict the formation and change of psychological safety

Deep-level diversity refers to underlying psychological characteristics, such as personality factors, abilities, and values (Bell, 2007). Team member personality characteristics and competencies have been identified as the most important predictors for effective teamwork, which is an important outcome associated with PS (Kozlowski and Bell, 2003; Newman et al., 2017). Team members’ personality characteristics translate to the team level, especially in self-managed teams where team members’ roles emerge and transform over time (Barry and Stewart, 1997). Compared to surface-level diversity, deep-level diversity regarding team member personality and competencies is found to be more important for team functioning over time (Harrison et al., 2002). The authors explain the finding in such a way that team members have to get to know each other first to learn about each other’s characteristics. On the other hand, the joint task may also reveal the requirements of certain characteristics at different points in time. In the current study, we refer to the five-factor model of personality, which is a widely accepted model of personality and is often called the Big Five (Goldberg, 1993; Costa and McCrae, 1994). It organizes broad individual differences in social and emotional experiences into five different categories—based on factor analyses applied to personality survey data (McAdams and Pals, 2006). These categories are usually labeled extraversion (vs. introversion), neuroticism (vs. emotional stability), openness to experience, conscientiousness, and agreeableness. The Big Five are relatively consistent and stable over time, have psychobiological underpinnings, and can predict behavioral tendencies and life outcomes (e.g., Matthews et al., 2009). They have also been used effectively in several past studies focused on individual differences between team members (e.g., Bonner et al., 2007).

Previous research links three of the Big Five personality characteristics, namely extraversion, openness to experiences, and neuroticism, to PS on a theoretical level (Edmondson and Mogelof, 2005). This research revealed that, on an individual level, neuroticism and openness to experience predict individual PS perceptions at the midpoint of a team project and that neuroticism predicts PS perceptions at the end of the project. Contrary to the authors’ expectations, extraversion did not predict PS perceptions at any measurement point. Edmondson and Mogelof (2005) explain the lack of an effect of extraversion by arguing that extraversion encompasses a wide variety of behaviors and thus, might not be close enough to PS-relevant behaviors such as speaking up—although extraversion is understood to be an important antecedent of this specific communication behavior. They proposed that the investigated personality characteristics extraversion, openness, and neuroticism might translate to the team level as well. Results from a team study on composition and compilation effects identified conscientiousness as an important predictor of PS if its distribution is skewed in the team (Ostermeier et al., 2020). Taken together, the theoretical link between personality characteristics and PS is promising and needs more clarity.

Despite the broader assumption that time catalyzes the effect of team personality (Harrison et al., 2002), we have reason to believe that extraversion and neuroticism are exceptions that affect the formation of PS. As such, extraversion is important for PS formation because individuals with high levels of extraversion enter new ambiguous social situations self-confidently (Anthony et al., 2007; Gebauer et al., 2015) and promote the experience of a positive mood in the team (Barrick et al., 1998). Neuroticism is important for PS formation, as it is closely linked to the concept of negative affectivity (Stokes and Levin, 1990), a dispositional tendency to experience aversive feelings, which has been shown to predict initial levels of PS (Ostermeier et al., 2020). Individuals with high levels of neuroticism and likewise teams with neurotic team members experience higher feelings of distress, anger, frustration, or anxiety in social interactions (Levin and Stokes, 1989) impairing the emergence of PS at the beginning and its development. Furthermore, deep-level diversity in terms of the personality characteristics of openness to experience, conscientiousness, and agreeableness should become more important during the later stages of group work when PS has developed further (Harrison et al., 2002).

We base our reasoning on the model proposed by LePine et al. (2011) on how team member personality characteristics can translate to the team level either through individual personality traits that directly affect team composition or corresponding behaviors that affect team processes indirectly. Following the principle of parsimony, we build our team research on two functional relations between team composition and PS proposing that team composition might have additive (when the combination of group members’ scores is equal to the sum of the effects) and/or compensatory effects (when group members’ scores on an attribute balance one another) regarding team characteristics. Thus, we relate team composition attributes with the respective team mean values and/or within-standard deviations as an index of variation to PS. We explain our reasoning in more detail below and postulate our assumptions on the team level linking team members’ personality characteristics to the formation and changes in PS.

In the current study, we focus on extraversion as a predictor for the formation of PS. Extraverts are sociable, enthusiastic, energetic, and assertive (Goldberg, 1993; Barrick et al., 1998). Behaviors relating to extraversion can be more easily observed than behaviors relating to the other Big Five (John and Robins, 1993). Therefore, extraversion should be particularly important at the beginning—as compared to the other traits. In teams with highly extroverted members, PS should form quickly when members signal to each other that the team has a friendly atmosphere where open communication is encouraged. This assumption is supported by a positive correlation between team extraversion (mean values) and communication on the team level (Barrick et al., 1998). Further, extraversion comprises the experience of positive emotional states (Barrick et al., 1998) that promote well-being and prepare the ground for a supportive learning atmosphere right from the start. This positive effect on PS is likely to be additive: the more extraverts belong to a team, the more signals of sociability and positive affectivity they might send to each other within the first meetings. If the team is high in extraversion (i.e., has a high mean in team member extraversion), the positive effect might wear out at a later stage of the collaboration when PS has been established. However, in the beginning, team members’ extraversion should facilitate communication behaviors and a positive sentiment toward each other that form the basis for PS. Thus, we propose:

*Hypothesis 3a_(E, Mean)_*: The higher a team’s mean level in extraversion, the higher the initial levels of psychological safety will be in the team.

The distribution of extraversion in the team should also be relevant to the formation of PS. Extraverts are assertive and tend to show leadership behaviors (Judge et al., 2002). Thus, if team members vary in their level of extraversion, some members might dominate other members from the start with a rather disconcerting, negative effect on other team members’ perception of the team environment for inter-individual risk-taking. Thus, we propose

*Hypothesis 3b_(E, SD)_*: The more diverse team members are in extraversion, the lower initial levels of psychological safety will be in the team.

As a second deep-level predictor for the formation and changes in PS, we chose neuroticism, which is closely linked with negative affectivity (Goldberg, 1993; Barrick et al., 1998), and should obstruct trust and communication behaviors that form the basis for PS. Team members who are high in neuroticism are less emotionally stable and tend to be anxious, depressive, and angry (George, 1990; Barrick et al., 1998). In contrast to their emotionally stable counterparts, highly neurotic team members should impede a continued process of positive interactions. More specifically, they should reduce the team’s responsiveness to feedback, because mistakes that are mentioned might be taken personally and not as constructive feedback. Team neuroticism should have an additive and a compensatory effect. A team composed of highly neurotic members is likely to create a tense atmosphere inhibiting cooperation, which translates into the joint perception that it is not safe to take risks from the beginning. A recent study about the relationship between negative affectivity and early PS supports this assumption: Teams high in negative affectivity, operationalized as a trait that reflects individuals’ dispositional tendency to experience aversive emotions, started teamwork with lower levels of PS (Ostermeier et al., 2020). Thus, we propose

*Hypothesis 3c_(N, Mean)_*: The higher a team’s mean level in neuroticism is, the lower initial levels of psychological safety will be.

In addition, diversity in neuroticism might become more important when mistakes occur, and task conflicts arise during project work. When team members are diverse in neuroticism, then there are a few members high in neuroticism; these members might destroy PS and impede team learning for everyone in the team by expressing their discomfort with feedback and open ideas. This effect can be explained by the positive–negative asymmetry, a phenomenon in impression formation that states that bad information carries more weight and influences the impression more than good information (e.g., Peeters and Czapinski, 1990). The underlying reason is that “bad” behaviors (in this case, the neurotics who express discomfort with feedback) are more diagnostic than “good” behaviors (in this case, the emotionally stable who express comfort with open feedback) because the consistency requirements for PS are more stringent than for the absence of PS. In other words, to experience PS, all team members must be comfortable with feedback. For a low level of PS, a few comments from neurotic team members are sufficient. Thus, we propose

*Hypothesis 3d_(N, SD)_*: The more diverse team members are in neuroticism, the more psychological safety will decrease over time.

As a third deep-level predictor for changes in PS, we chose openness to experience, because it is closely linked with learning behaviors. Open individuals are naturally curious and open-minded regarding unconventional ideas and experimentation (Goldberg, 1993; Barrick et al., 1998). As such, teams with high levels of openness are likely to foster the discussion of problems by having members offer unconventional ideas or inviting each other to think outside the box. This kind of communication is more important during collaboration—and especially when teams encounter difficulties—than at the beginning. Translating the relationship between openness to experience and PS found by Edmondson and Mogelof (2005) to the team level, we postulate an additive effect of team member openness on the development of PS:

*Hypothesis 3e_(O, Mean)_*: The higher a team’s mean level in openness to experience, the more psychological safety within the team will increase over time.

As the fourth deep-level predictor for PS, we chose conscientiousness, an important characteristic for thorough work, organization, and the detection of errors (Goldberg, 1993; Barrick et al., 1998) that can be linked to specific learning behaviors. A team composed of highly conscientious members works thoroughly, is achievement-oriented, and engages in task-focused roles (Bell, 2007). Especially the aspect of duty fulfillment is an important part of the concept of conscientiousness that should foster PS in teams because members have a high interest in doing the right thing for themselves and others. They understand that sharing relevant information is part of their duty that might create a norm on the team level (Ostermeier et al., 2020). Further, conscientiousness is related to helping behavior in a consistent way (Hurtz and Donovan, 2000). In a recent study, team mean conscientiousness was hypothesized to have a positive effect on PS (Ostermeier et al., 2020). Yet, results from a path model did not support this assumption. Instead, team conscientiousness was found to have a negative effect on PS, if its distribution was skewed in the team (Ostermeier et al., 2020). More specifically, if conscientiousness was positively skewed in a team, that is, when there were more highly conscientious members compared to members low in conscientiousness, PS decreased. Based on these findings, we propose the following

*Hypothesis 3f_(C, SD)_*: The more diverse team members are in conscientiousness, the more psychological safety will decrease over time.

Finally, as the fifth deep-level predictor for PS, we chose agreeableness. It describes characteristics such as “helpful, friendly, warm, trusting, and tolerant. In fact, the very essence of agreeableness is cooperation” (Barrick et al., 1998, p. 381). We find a theoretical overlap between the concepts of agreeableness and PS, as it encompasses attributes of a comfortable learning atmosphere such as trustful, warm, tolerant, as well as specific learning behaviors such as being helpful, considerate, or caring. A team composed of highly agreeable members might encompass a group norm of being prosocial and inclined toward others (Graziano et al., 2007). Such teams report having fewer conflicts (Barrick et al., 1998). A recent study investigated the relation between team agreeableness and initial levels and changes in cohesion—the forces that help members belong to the team (Festinger, 1950). Acton et al. (2020) found that teams composed of highly agreeable members reported an increase in cohesion over time. PS and cohesion are both emergent states, only PS fosters the exchange of divergent opinions (Edmondson, 2002), whereas cohesion is partly related to speaking up less often and exchanging opposing opinions less strongly (Rose et at., 2011). Based on these findings, we propose

*Hypothesis 3g_(A, Mean)_*: The higher a team’s mean level of agreeableness, the more psychological safety within the team will increase over time.

Still, a single highly disagreeable member can be enough to undermine the team’s capability to work together (Barrick et al., 1998). When members of a team strongly vary in their agreeableness, this can damage the relationships between team members. For instance, a disagreeable team member might start conflict stressing agreeable members in their striving for harmony and a task-focused working atmosphere at a later point of collaboration. The effect of variability in team member agreeableness can also be explained by the positive–negative asymmetry (e.g., Peeters and Czapinski, 1990). The “bad” behaviors of disagreeable team members who start conflicts have a stronger impact on PS as compared to the “good” behaviors of agreeable team members striving for harmonious interactions. In previous research, variance in team agreeableness was negatively related to social cohesion, communication, and workload sharing, while being positively related to team conflict (Barrick et al., 1998). We therefore propose.

*Hypothesis 3h_(A, SD)_*: The more diverse team members are in agreeableness, the more psychological safety will decrease over time.

Besides team member personality characteristics, team member competencies should also predict the development of PS. Member competencies are conceptualized as personal characteristics or specific behaviors that are related to performance in a certain context (Krumm et al., 2012). We refer to task-relevant competencies as an important resource for team learning because they help members identify and correct mistakes and encourage them to consult each other for advice (Doblinger, 2022). They influence what and how team members communicate and how the team operates and performs (Hackman et al., 1976). Teams need to generate performance strategies that are appropriate to solve the task, and they do so based on their knowledge and competencies (Hackman et al., 1976). However, a high level of competency does not necessarily need to translate into positive results. On the contrary, members generate private hypotheses about how a task needs to be approached or solved and rarely discuss it explicitly with each other (Hackman et al., 1976). Task-oriented teams tend to meet the implicit norm of NOT addressing process issues (Argyris, 1969), which means that team members share less or no information about their individual strategies. In an experiment with student teams (Hackman et al., 1976), teams with unequal information about the task performed better, if an explicit discussion of performance strategies was a part of their team task (as compared to teams in the control group without intervention or team in another intervention group that prohibited the discussion of performance strategies as inefficient and waste of time). Teams with equal information only performed well when they did not discuss performance strategies at the beginning of the team task. Previous research supports the current relevance of this phenomenon, showing that teams differ in their ability to utilize their resources and successfully transform them into good team decisions, team performance, or creativity (Innami, 1994; Taggar, 2002; Mayo and Woolley, 2021). The reason for this unexpected negative link lies in the teams’ communication behavior. Teams with ample resources (such as high task-relevant knowledge) tend to have discussions with members sticking to their positions and exchanging fewer facts and reasons to explain their positions as compared to teams with little resources (Innami, 1994). This finding is in line with former observations as described above (e.g., Argyris, 1969; Hackman et al., 1976), thereby relating team members’ competencies to specific learning behaviors, namely exploring new knowledge and information and engaging in a collaborative conversation to refine, build or modify collective knowledge (Wiese and Burke, 2019). Based on these previous findings, we believe that team member competencies can also impact PS perceptions. More specifically, they should impact how PS changes over time. Team member competencies should have an additive effect, that is, teams with high task-relevant competencies might be more likely to detect problems or provide solutions, yet, they might have difficulties elaborating on adequate solutions due to their reduced communication behavior (Innami, 1994). Thus, we propose

*Hypothesis 4_(Competency, Mean)_*: Team member task-relevant competencies predict changes in psychological safety: The greater a team’s competency, the more psychological safety will decrease over time.

## Materials and methods

### Participants

Our study was part of a bigger research project about time and changes in teams at a university in Germany. Sixty-one student teams of two to five members each participated in this study (*M* = 3.87, *SD* = 0.53). They worked on a university research project, which was part of their curriculum, over the course of 5 months. In total, 236 students participated in this study. Of these students, 68% were psychology students and 32% were students from a bachelor’s program combining psychology, physics, and cognitive sciences. Data were collected over a period of three semesters. The majority of the participants were female (77.12%) and in their second year of study. Participants were between 19 and 44 years old (*M* = 22.34, *SD* = 3.43).

### Team task and participant recruitment

In their second year of study, psychology students needed to complete a course in experimental research. Their task was to design and conduct a research project: They developed hypotheses, recruited participants, collected data, ran analyses, and critically discussed their results. At the first course meeting, we invited students to participate in our study. At this time, some teams had already formed on a voluntary basis. We briefly described the goal of our study and explained how data would be collected. In exchange for every completed questionnaire, students received research credits. In addition, teams could win a movie voucher in a lottery to celebrate their project accomplishment. All 62 teams who were invited to the study participated. We excluded one team consisting of four members from further analyses because one member quit the class after the midpoint of the project leaving team assessments of PS incomplete.

### Design and procedure

The longitudinal design makes it possible to study temporal dynamics in teams. We asked team members to complete questionnaires at three points in time that are important for team development, namely at the beginning, at the midpoint, and at the end of the project (Gersick, 1988; Chang et al., 2003). We further related our measurement points to the following tasks that teams had to fulfill during their course: at the first point of measurement, approximately 4 weeks following the start of the class, teams presented the theoretical framework for their projects. We assumed that team members required this time to meet and get acquainted with each other before PS could be assessed adequately. At the second point of measurement, when half of the project time had passed, teams presented their methods. At the third point of measurement, which was at the end of the project, teams presented and discussed their results. Data were collected anonymously. Team diversity variables were assessed at the first measurement point. Team members assessed their PS at all three measurement points.

### Measures

All scales had the same response format: On a Likert-type scale, team members rated the extent to which they agreed with the statements, ranging from 1 (*not at all*) to 7 (*absolutely*).

#### Team psychological safety

We assessed PS with the Team Psychological Safety Scale (Edmondson, 1999). An example item is “If you make a mistake on this team, it is often held against you” (reverse scored). We translated the scale into German, remaining as close to the original expressions as possible. After the first point of measurement, several team members reported having difficulties in responding to the fourth item “It is safe to take a risk on this team.” We excluded this item so that six items remained in our analyses (We report omega total as suggested by McNeish, 2018 and Hayes and Coutts, 2020: *ω_total1_* = 0.59, *ω_total2_* = 0.77, and *ω_total3_* = 0.70 for six items each).

#### Demographic faultline strength

We calculated demographic faultline strength using the average silhouette width (ASW) cluster algorithm in R (Meyer and Glenz, 2013). The surface-level faultline included the demographic characteristics of gender, age, course of study (psychology or cognitive science), and semester term (third, fourth, or fifth semester).

The ASW measure lies between −1 and 1. Values near 1 represent the emergence of mostly homogeneous subgroups, whereas values near 0 represent mostly diverse subgroups as no homogeneous subgroups exist. ASW categorizes members into subgroups in a stepwise approach. First, each team member has his or her own subgroup, consisting of him or herself only. Next, members with the most similarities are merged into a subgroup of two, and so on, until all existing subgroup possibilities are calculated. Then, the mean ASW value is computed for each team member representing how well the member fits into the subgroup. The output in R provides faultline values per team and further information on the team level such as the number of subgroups, the subgroup size, and an explicit allocation of members to subgroups (see Meyer et al., 2015). In most teams (*n* = 52), only two homogeneous subgroups emerged. The subgroup size was, on average, 2.2 members per subgroup.

#### Team member personality

We used the Big Five Inventory-25 to measure the personality dimensions of extraversion, neuroticism, openness to experience, conscientiousness, and agreeableness with five items each. The inventory is a shorter version of the 44-item Big Five Inventory by John et al. (1991), which was translated into German and validated by Rammstedt et al. (2004). Example items are “I see myself as someone who is “…talkative” for extraversion (*ω_total1_* = 0.90), “… worries a lot” for neuroticism (*ω_total1_* = 0.84), “…inventive” for openness (*ω_total1_* = 0.86), “… tends to be disorganized” (inverted item) for conscientiousness (*ω_total1_* = 0.84), and “… generally trusting” for agreeableness (*ω_total1_* = 0.69).

#### Team member competency

We assessed team members’ competencies in statistics and methodology because group members needed them in all steps of their empirical research project. For example, when reading the introductory articles, they needed to understand what had been done before and how. When planning the experiment, basic methodological knowledge helped to select a design and appropriate measures. When analyzing data, statistical knowledge is essential for testing hypotheses. We adapted a German version of the Academic Self-Description Questionnaire (ASDQ; Marsh et al., 2005b) to the context of these groups. In general, the ASDQ measures self-assessed competencies in school subjects with six items each (e.g., in math, language, or sports). We replaced the school subjects with statistics and methodology. An example item is “In comparison to my fellow students, I am very good at statistics and methodology” (*ω_total1_* = 0.90). Marsh et al. (2005a) reported moderate correlations between ASDQ assessments and the respective grades. Because students had taken courses in statistics before participating in this group research project, we assumed that they were able to properly answer the questionnaire.

#### Control variables

When testing the effects of demographic faultlines, we included demographic attributes that may affect the team outcome above and beyond faultline strength on the team level (see Meyer et al., 2015). We operationalized demographic diversity effects on a team level by including the Blau index (Blau, 1977) for gender diversity and the within-team standard deviation in tenure (number of semesters).

## Results

We used multilevel-based linear growth curve modeling (LGCM) to test our hypotheses (Gałecki and Burzykowski, 2013; Bliese, 2016). Linear growth curve analysis is used in several psychological disciplines to investigate developmental trajectories, e.g., those of personality development or the impact of time on the development of social behavior (Jung and Wickrama, 2008). This method allows us to study the development of PS in the form of overall increase or decrease by modeling initial levels (the intercept) and changes within teams (interaction with time). With this statistical method, we can examine differences within and between teams over time and we can test our hypotheses about how team faultlines and diversity in member personality and competencies predict initial levels and changes in PS.

### Agreement between team members and data aggregation

In the first step, we calculated agreements between team members to test for the nested structure of our data (Bliese, 2016). Thus, we calculated intraclass correlation coefficients *ICC(1)* and *ICC(2)*. The *ICC(1)* value indicates the amount of variance that can be explained by team membership. The *ICC(2)* value indicates the extent to which the team’s mean rating is reliable, thereby taking average team size into account (Bliese, 2000; Grawitch and Munz, 2004). As expected, we found high and significant *ICC* values for PS, with *ICC(1)* ranging between 0.14 and 0.26, and *ICC(2)* between 0.37 and 0.58 across measurement points. The findings indicate high agreements within teams and differences between teams. In comparison, we found non-significant *ICC* values for four of the five personality characteristics and competencies indicating differences between team members, with *ICC(1)* ranging between 0 and 0.08, and *ICC(2)* between.0 and 0.22; except for conscientiousness with *ICC(1)* = 0.12, *p* < 0.05 and *ICC(2)* = 0.35. Because we were interested in how team diversity variables, and not individual characteristics, affect initial assessments and changes in PS, we aggregated all predictor variables to the team level for hypothesis testing. We used the standard deviation within teams as an index for team diversity regarding team member personality (see Harrison et al., 2002). We also tested for measurement invariance (MI) of the PS Scale before conducting growth analysis as recommended by Van de Schoot et al. (2012). Results confirmed a strict invariance of the PS scale across the three measurement points because the models had a very good fit and did not differ significantly from one another (configural MI, metric MI, adjusted scalar MI, and strict MI). Correlations between demographic faultline strength, team diversity variables, and team PS are displayed in Table 1.

**Table 1:** Means, SD, and correlations among variables at team level at all measurement points (*N* = 61 teams).

Variable	*M*	*SD*	1.1	1.2	1.3	1.4	1.5_E_	1.5_N_	1.5_O_	1.5_C_	1.5_A_	1.6	1.7_E_	1.7_N_	1.7_O_	1.*7_C_*	1.7_A_	1.8	1.9	2	3
Beginning																					
1.1 Team Size	3.87	0.53	**–**																		
Surface-Level Diversity																					
1.2 Gender, Blau’s Index	0.21	0.27	−0.13	**–**																	
1.3 Tenure, *S.D.*	0.28	0.65	−0.20	**0.26**	**–**																
1.4 Demographic Faultline Strength	0.51	0.18	**0.41**	−0.22	0.14	**–**															
Deep-Level Diversity																					
1.5_E_ Extraversion, *Mean*	4.57	0.68	0.25	0.01	**−0.32**	0.09	**–**														
1.5_N_ Neuroticism, *Mean*	4.29	0.61	−0.03	−0.24	−0.13	0.02	−0.22	**–**													
1.5_O_ Openness to Experience, *Mean*	5.30	0.50	0.05	0.07	0.08	−0.05	0.11	**−0.39**	**–**												
1.5_C_ Conscientiousness, *Mean*	5.31	0.66	0.08	**−0.49**	−0.09	0.17	0.12	0.18	−0.10	**–**											
1.5_A_ Agreeableness, *Mean*	5.70	0.39	0.00	−0.18	0.16	0.04	0.08	0.05	0.15	**0.34**	**–**										
1.6 Competency, *Mean*	4.43	0.74	−0.03	**0.27**	−0.11	−0.19	0.16	−0.02	0.01	−0.15	−0.14	**–**									
1.7_E_ Extraversion, *S.D.*	1.23	0.52	0.21	−0.03	−0.07	−0.00	−0.03	0.03	0.02	0.08	−0.13	−0.02	**–**								
1.7_N_ Neuroticism, *S.D.*	1.08	0.50	0.10	0.23	−0.22	0.12	0.13	0.04	0.02	−0.06	0.00	0.08	0.07	**–**							
1.7_O_ Openness to Experience, *S.D.*	0.93	0.46	0.11	−0.07	−0.14	0.05	0.25	0.06	**−0.45**	0.06	−0.15	0.02	0.14	0.04	**–**						
1.7_C_ Conscientiousness, *S.D.*	0.91	0.42	**0.33**	0.04	0.23	0.21	−0.01	0.21	0.02	0.05	0.09	−0.04	0.15	−0.10	0.05	**–**					
1.7_A_ Agreeableness, *S.D.*	0.77	0.30	−0.00	0.05	0.05	0.14	−0.08	0.13	−0.04	−0.07	**−0.30**	0.08	−0.13	0.12	0.05	**0.27**	**–**				
1.8 Competency, *S.D.*	1.13	0.47	0.25	−0.17	0.08	**0.30**	−0.06	−0.08	0.21	0.19	0.02	−0.14	0.14	0.02	0.11	**0.34**	**0.30**	**–**			
Team Emergent State																					
1.9 Team Psychological Safety	6.01	0.41	−0.14	0.01	0.08	**−0.26**	0.10	−0.17	0.20	0.13	0.19	−0.03	0.02	**−0.32**	−0.09	−0.20	0.06	0.05	**–**		
Midpoint																					
2 Team Psychological Safety	5.85	0.52	−0.16	−0.03	−0.06	−0.22	0.18	−0.06	0.06	0.09	0.06	−0.05	−0.02	−0.24	−0.01	−0.13	0.01	0.01	**0.58**	**–**	
End																					
3 Team Psychological Safety	5.81	0.56	−0.09	−0.14	−0.12	−0.22	0.09	−0.04	0.13	**0.29**	0.23	**−0.26**	0.08	−0.00	−0.15	−0.18	−0.19	0.06	**0.52**	**0.66**	**–**

*M* and *SD* are used to represent mean and standard deviation, respectively. Correlations with *r* > 0.25 are significant at *p* < 0.05 and are bold printed.

### The reasoning of control variables

Due to the complexity of our tested model, we reduced control variables to the least possible number according to their theoretical contribution to our model (Becker, 2005). First, we controlled for diversity regarding gender, because women differ in their perceptions of PS as compared to men (Carmeli and Gittell, 2009). Second, we controlled for tenure diversity. Tenure has a distinct meaning for personal and organizational identity building as compared to age, especially if members enter the organization at the same time (e.g., Meyer et al., 2015). Faultline attributes often correlate with each other, such as age and tenure. However, as they share the same variance, they might shadow possible effects due to multicollinearity. Because the difference in team members’ theoretical knowledge of psychology in terms of their tenure was more important for the successful completion of the task than their age differences, we included tenure (semester term) in our final model calculations.

Finally, our teams were all relatively small (with 3–5 members each and one dyad), and team size did not correlate with PS. Because team faultlines and team size correlated significantly, we excluded team size as a control variable when testing our hypotheses referring to initial levels of PS. When testing for changes in PS, however, we included team size again, because decisions and conflicts might differ in teams with three members compared to four or five members, when majorities and coalitions can be formed.

### Analytical procedure

We used Linear Growth Curve Modeling in R (R Development Core Team, 2012), a multilevel approach that allows us to study changes (Bliese and Ployhart, 2002; Bliese, 2016). This way, we can predict initial levels (the intercepts) and changes (the slopes) of PS over time. We had two levels of analysis with measurement points on the lowest level (level 1), nested in teams (level 2). We restructured the dataset according to a new variable, namely Measurement Time with the attributes 1 (initial levels), 2 (midpoint), and 3 (end). In our model-building approach (Bliese and Ployhart, 2002), we started by calculating a null model and increased the complexity of the models in a stepwise way.

In the first step, we examined the base growth curve and fitted the model’s structure. In the second step, we predicted initial levels of PS by adding demographic faultline strength as a focal predictor to the model, when controlling for the components of the faultlines, namely diversity in gender and tenure. In the third step, we predicted initial levels of PS by adding deep-level diversity in terms of personality (mean values and SDs) for extraversion and neuroticism step by step to the model, again controlling for diversity in gender and tenure. In the fourth step, we predicted changes in PS by adding deep-level diversity in terms of the hypothesized personality characteristics and competencies (mean values and SDs) with the respective interactions with time to the baseline model, when controlling for team size as well as diversity in gender and tenure. When testing our hypotheses, we added both the mean and the within-team deviation of the deep-level predictors to the model to accurately consider their dependency when interpreting the results (e.g., we combined mean values and controlled for deviation, and vice versa). All predictors on the team level were grand-mean centered, except for demographic faultline strength and team diversity regarding gender, which were *z*-standardized.

### Fitting the model’s structure

First, we ran an unconstrained (null) model and found confirmation for our multilevel structure. In this model, 25% of the variance in PS could be explained by team properties. Second, we calculated a random intercept, fixed slope model by adding measurement time, a level-1 variable, to the model. A comparison of the models’ deviations showed that a random intercept, fixed slopes model had a significantly better fit compared to the null model [*Δ-2LL* (1) = 11.71, *p* < 0.0006]. In the next step, we tested a random intercept, random slopes model, allowing slopes to vary as well. Again, this model had a significantly better fit compared to the former model [*Δ-2LL* (5) = 26.30, *p* < 0.0001]. Third, we tested for autocorrelation among measurement points. This model had no significantly better fit compared to the former model [*Δ-2LL* (1) = 0.01, *p* = 0.93]. Therefore, we allowed intercepts and slopes to vary in further calculations without controlling for autocorrelation in our data. We report our base growth curve model as Model 1 (Table 2).

**Table 2:** Linear growth curve model (*n* = 699 Measurements Nested in Three Time Points, in 233 Members in 61 Teams) for initial levels of team psychological safety.

*Parameter*	*Model 1_Time_*			*Model 2_Faultlines_*			*Model 3_Extraversion_*			*Model 4_Neuroticism_*			*Model 5_Combined_*		
	*b*	*95% CI*	*t*	*b*	*95% CI*	*t*	*b*	*95% CI*	*t*	*b*	*95% CI*	*t*	*b*	*95% CI*	*t*
**Fixed Effects Level 1 (Time)**															
Intercept	**6.09**	[5.96, 6.22]	91.30	**6.09**	[5.96, 6.22]	90.42	**6.09**	[5.96, 6.23]	91.30	**6.09**	[5.97, 6.22]	96.35	**6.09**	[5.97, 6.21]	96.46
Time	**−0.09**	[−0.15, −0.03]	−3.09	**−0.09**	[−0.15, −0.03]	−3.11	**−0.09**	[−0.15, −0.03]	−3.09	**−0.09**	[−0.15, −0.03]	−3.08	**−0.09**	[−0.15, −0.03]	−3.10
**Fixed Effects Level 2 (Teams)**															
Surface-Level Diversity															
Gender, Blau’s Index				−0.04	[−0.15, 0.07]	−0.76	−0.01	[−0.12, 0.09]	−0.23	0.02	[−0.09, 0.13]	0.41	−0.02	[−0.13, 0.10]	−0.32
Tenure, *S.D.*				0.06	[−0.12, 0.23]	0.64	0.06	[−0.12, 0.24]	0.70	−0.05	[−0.22, 0.13]	−0.53	0.05	[−0.14, 0.24]	0.55
Demographic Faultline Strength				**−0.11**	[−0.21, −0.002]	−2.03							−0.09	[−0.20, 0.01]	−1.72
Team Personality															
Extraversion, *Mean*							0.12	[−0.04, 0.29]	1.53				0.14	[−0.03, 0.30]	1.69
Extraversion, *S.D.*							0.06	[−0.14, 0.26]	0.62				0.07	[−0.12, 0.26]	0.70
Neuroticism, *Mean*										−0.11	[−0.27, 0.06]	−1.29	−0.08	[−0.25, 0.09]	−0.95
Neuroticism, *S.D.*										**−0.25**	[−0.46, −0.04]	−2.42	**−0.22**	[−0.44, −0.01]	−2.10
Variance Components															
Level 2 Intercept (Teams)	**0.33**	[0.20, 0.52]		0.33			0.33			**0.28**	[0.16, 0.50]		0.28		
Level 2 Slope (Teams)	**0.17**	[0.12, 0.24]		0.17			0.17			**0.17**	[0.12, 0.24]		0.17		
Intercept-Slope Covariance (Teams)	**−0.61**	[−0.85, −0.16]		−0.61			−0.60			**−0.57**	[−0.85, −0.04]		−0.59		
Level 1 Intercept (Time)	**0.42**	[0.31, 0.55]		0.42			0.43			**0.43**	[0.30, 0.63]		0.43		
Level 1 Slope (Time)	**0.05**	[0.01, 0.21]		0.06			0.06			**0.06**	[0.005, 0.71]		0.06		
Intercept-Slope Covariance (Time)	0.74	[−0.96, 0.999]		0.55			0.53			0.47	[−0.99, 0.999]		0.46		
Within-Team Variance	**0.44**	[0.41, 0.48]		0.43			0.43			**0.43**	[0.38, 0.50]		0.43		
*AIC*	1294.68			1305.53			1315.26			1310.33			1316.18		
*BIC*	1335.37			1364.20			1378.48			1373.56			1392.80		
*– 2LL*	1276.67			1279.53			1287.26			1282.33			1282.19		
Overall Pseudo-*R^2^_m_*	0.01			0.03			0.02			0.04			0.07		

All models are random intercepts, random slopes, and – 2 ll = − 2 log likelihood values. For Models 2, 3 and 5, only confidence intervals for fixed effects are reported; confidence intervals could not be calculated in R due to a non-positive definite in the variance–covariance matrix. Values significant according to 95% CI are bold.

### Hypothesis tests

All tested models with the respective coefficients, variances and fit indices are presented in tables. Table 2 shows the test of predictors for initial levels in PS, and Tables 3-6 show the tests of predictors for changes in PS. Table 7 presents an overview of the tested hypotheses and respective support.

**Table 3:** Linear Growth Curve Model (*n* = 699 measurements nested in three time points, in 233 members in 61 teams) for changes in team psychological safety predicted by team personality and competency (Models 6 and 7).

*Parameter*	*Model 6*			*Model 7_Neuroticism_*		
	*b*	*95% CI*	*t*	*b*	*95% CI*	*t*
Fixed Effects Level 1 (Time)						
Intercept	**6.09**	[5.95, 6.23]	89.80	**6.09**	[5.97, 6.22]	95.69
Time	**−0.09**	[−0.15, −0.03]	−3.10	**−0.09**	[−0.15, −0.03]	−3.12
Fixed Effects Level 2 (Teams)						
Team Size	−0.08	[−0.29, 0.13]	−0.77	−0.06	[−0.26, 0.14]	−0.63
Surface-Level Diversity						
Gender, Blau’s Index	−0.01	[−0.12, 0.10]	−0.18	0.02	[−0.09, 0.13]	0.30
Tenure, *S.D.*	−0.003	[−0.17, 0.18]	0.04	−0.05	[−0.23, 0.12]	−0.60
Big Five and Team Competency						
Neuroticism, *Mean*				−0.13	[−0.35, 0.08]	−1.25
Openness to Experience, *Mean*						
Conscientiousness, *Mean*						
Agreeableness, *Mean*						
Competency, *Mean*						
Neuroticism, *S.D.*				**−0.39**	[−0.66, −0.12]	−2.93
Openness to Experience, *S.D.*						
Conscientiousness, *S.D.*						
Agreeableness, *S.D.*						
Competency, *S.D.*						
Cross-Level Interactions						
Time × Neuroticism, *Mean*				0.02	[−0.08, 0.11]	0.35
Time × Openness to Experience, *Mean*						
Time × Conscientiousness, *Mean*						
Time × Agreeableness, *Mean*						
Time × Competency, *Mean*						
Time × Neuroticism, *S.D.*				0.11	[−0.01, 0.23]	1.78
Time × Openness to Experience, *S.D.*						
Time × Conscientiousness, *S.D.*						
Time × Agreeableness, *S.D.*						
Time × Competency, *S.D.*						
Variance Components						
Level 2 Intercept (Teams)	**0.34**	[0.22, 0.54]		**0.29**	[0.17, 0.50]	
Level 2 Slope (Teams)	**0.17**	[0.12, 0.24]		**0.17**	[0.12, 0.24]	
Intercept-Slope Covariance (Teams)	**−0.59**	[−0.84, −0.15]		**−0.56**	[−0.84, −0.05]	
Level 1 Intercept (Time)	**0.43**	[0.32, 0.59]		**0.43**	[0.30, 0.62]	
Level 1 Slope (Time)	**0.06**	[0.01, 0.39]		**0.06**	[0.01, 0.48]	
Intercept-Slope Covariance (Time)	0.45	[−0.93, 0.99]		0.75	[−0.988, 0.999]	
Within-Team Variance	**0.43**	[0.38, 0.49]		**0.43**	[0.37, 0.50]	
*AIC*	1311.98			1323.27		
*BIC*	1370.71			1399.97		
*–2LL*	1285.98			1289.27		
Overall Pseudo-*R^2^_m_*	0.01			0.04		

*Note*. All models are random intercepts, random slopes, and −2 LL = −2 log likelihood values. Values significant according to *95% CI* are bold.

**Table 4:** Linear Growth Curve Model (*n* = 699 measurements nested in three time points, in 233 members in 61 teams) for changes in team psychological safety predicted by team personality and competency (Models 8 and 9).

*Parameter*	*Model 8*_Openness_			*Model 9*_Conscientiousness_		
	*b*	*95% CI*	*t*	*b*	*95% CI*	*t*
Fixed Effects Level 1 (Time)						
Intercept	**6.09**	[5.96, 6.23]	90.52	**6.09**	[5.96, 6.22]	89.83
Time	**−0.09**	[−0.15, −0.04]	−3.12	**−0.09**	[−0.15, −0.03]	−3.11
Fixed Effects Level 2 (Teams)						
Team Size	−0.09	[−0.30, 0.12]	−0.89	−0.02	[−0.24, 0.20]	−0.18
Surface-Level Diversity						
Gender, Blau’s Index	−0.02	[−0.12, 0.09]	−0.30	0.03	[−0.09, 0.15]	0.47
Tenure, *S.D.*	−0.01	[−0.18, 0.17]	−0.07	0.04	[−0.14, 0.22]	0.41
Big Five and Team Competency						
Neuroticism, *Mean*						
Openness to Experience, *Mean*	0.25	[−0.06, 0.55]	1.63			
Conscientiousness, *Mean*				0.002	[−0.23, 0.23]	0.02
Agreeableness, *Mean*						
Competency, *Mean*						
Neuroticism, *S.D.*						
Openness to Experience, *S.D.*	0.15	[−0.19, 0.49]	0.86			
Conscientiousness, *S.D.*				−0.18	[−0.53, 0.17]	−1.04
Agreeableness, *S.D.*						
Competency, *S.D.*						
Cross-Level Interactions						
Time × Neuroticism, *Mean*						
Time × Openness to Experience, *Mean*	−0.04	[−0.18, 0.09]	−0.65			
Time × Conscientiousness, *Mean*				0.08	[−0.01, 0.17]	1.65
Time × Agreeableness, *Mean*						
Time × Competency, *Mean*						
Time × Neuroticism, *S.D.*						
Time × Openness to Experience, *S.D.*	−0.09	[−0.24, 0.06]	−1.18			
Time × Conscientiousness, *S.D.*				−0.02	[−0.16, 0.13]	−0.22
Time × Agreeableness, *S.D.*						
Time × Competency, *S.D.*						
Variance Components						
Level 2 Intercept (Teams)	**0.33**	[0.21, 0.53]		**0.34**	[0.21, 0.54]	
Level 2 Slope (Teams)	**0.17**	[0.12, 0.25]		**0.17**	[0.12, 0.24]	
Intercept-Slope Covariance (Teams)	−**0.60**	[−0.85, 0.15]		**−0.62**	[−0.85, 0.18]	
Level 1 Intercept (Time)	**0.43**	[0.31, 0.59]		**0.43**	[0.32, 0.57]	
Level 1 Slope (Time)	**0.06**	[0.01, 0.42]		**0.06**	[0.01, 0.26]	
Intercept-Slope Covariance (Time)	0.52	[−0.98, 0.998]		0.52	[−0.86, 0.98]	
Within-Team Variance	**0.43**	[0.39, 0.49]		**0.44**	[0.39, 0.48]	
*AIC*	1327.67			1326.38		
*BIC*	1404.37			1403.08		
*–2LL*	1293.67			1292.38		
Overall Pseudo-*R^2^_m_*	0.03			0.04		

*Note*. All models are random intercepts, random slopes, and −2 LL = −2 log likelihood values. Values significant according to *95% CI* are bold.

**Table 5:** Linear Growth Curve Model (*n* = 699 measurements nested in three time points, in 233 members in 61 teams) for changes in team psychological safety predicted by team personality and competency (Models 10 and 11).

*Parameter*	*Model 10_Agreeableness_*			*Model 11_Competency_*		
	*b*	95% CI	*t*	*b*	95% CI	*t*
Fixed Effects Level 1 (Time)						
Intercept	**6.09**	[5.96, 6.22]	90.54	**6.09**	[5.96, 6.23]	88.67
Time	**−0.09**	[−0.15, −0.04]	−3.17	**−0.09**	[−0.15, −0.04]	−3.18
Fixed Effects Level 2 (Teams)						
Team Size	−0.09	[−0.30, 0.12]	−0.84	−0.09	[−0.31, 0.13]	−0.85
Surface-Level Diversity						
Gender, Blau’s Index	0.007	[−0.10, 0.12]	0.13	0.003	[−0.11, 0.12]	0.07
Tenure, *S.D.*	−0.03	[−0.21, 0.15]	−0.31	−0.02	[−0.20, 0.17]	−0.17
Big Five and Team Competency						
Neuroticism, *Mean*						
Openness to Experience, *Mean*						
Conscientiousness, *Mean*						
Agreeableness, *Mean*	0.18	[−0.20, 0.56]	0.93			
Competency, *Mean*				0.07	[−0.13, 0.27]	−0.72
Neuroticism, *S.D.*						
Openness to Experience, *S.D.*						
Conscientiousness, *S.D.*						
Agreeableness, *S.D.*	0.39	[−0.09, 0.87]	1.64			
Competency, *S.D.*				0.02	[−0.29, 0.33]	0.12
Cross-Level Interactions						
Time × Neuroticism, *Mean*						
Time × Openness to Experience, *Mean*						
Time × Conscientiousness, *Mean*						
Time × Agreeableness, *Mean*	0.03	[−0.13, 0.18]	0.31			
Time × Competency, *Mean*				**−0.08**	[−0.16, −0.001]	−1.98
Time × Neuroticism, *S.D.*						
Time × Openness to Experience, *S.D.*						
Time × Conscientiousness, *S.D.*						
Time × Agreeableness, *S.D.*	**−0.21**	[−0.41, −0.0001]	−1.97			
Time × Competency, *S.D.*				0.01	[−0.11, 0.14]	0.23
Variance Components						
Level 2 Intercept (Teams)	0.33			0.35		
Level 2 Slope (Teams)	0.16			0.17		
Intercept-Slope Covariance (Teams)	−0.57			−0.59		
Level 1 Intercept (Time)	0.43			0.43		
Level 1 Slope (Time)	0.06			0.06		
Intercept-Slope Covariance (Time)	0.54			0.53		
Within-Team Variance	0.44			0.44		
*AIC*	1322.75			1329.18		
*BIC*	1399.45			1405.88		
*−2LL*	1288.75			1295.18		
Overall Pseudo-*R^2^_m_*	0.03			0.03		

*Note*. All models are random intercepts, random slopes models, −2 LL = −2 log likelihood values. For Models 10 and 11, only confidence intervals for fixed effects are reported; confidence intervals for the variance components could not be calculated in *R* due to a non-positive definite in the variance-covariance matrix. Values significant according to *95% CI* are bold.

**Table 6:** Linear Growth Curve Model (*n* = 699 measurements nested in three time points, in 233 members in 61 teams) for changes in team psychological safety predicted by team personality and competency (Model 12).

*Parameter*	*Model 12_Combined_*		
	*b*	*95% CI*	*t*
Fixed Effects Level 1 (Time)			
Intercept	**6.09**	[5.97, 6.22]	96.24
Time	**−0.09**	[−0.15, −0.04]	−3.27
Fixed Effects Level 2 (Teams)			
Team Size	−0.04	[−0.26, 0.19]	−0.33
Surface-Level Diversity			
Gender, Blau’s Index	0.08	[−0.05, 0.21]	1.27
Tenure, *S.D.*	−0.07	[−0.27, 0.12]	−0.75
Big Five and Team Competency			
Neuroticism, *Mean*	−0.07	[−0.32, 0.18]	−0.56
Openness to Experience, *Mean*	0.22	[−0.11, 0.56]	1.35
Conscientiousness, *Mean*	0.03	[−0.20, 0.26]	0.26
Agreeableness, *Mean*	0.25	[−0.13, 0.64]	1.32
Competency, *Mean*	0.05	[−0.14, 0.23]	0.51
Neuroticism, *S.D.*	**−0.40**	[−0.68, −0.13]	−2.94
Openness to Experience, *S.D.*	0.18	[−0.16, 0.52]	1.08
Conscientiousness, *S.D.*	−0.15	[−0.52, 0.22]	−0.79
Agreeableness, *S.D.*	0.46	[−0.04, 0.97]	1.86
Competency, *S.D.*	−0.07	[−0.41, 0.26]	−0.43
Cross-Level Interactions			
Time × Neuroticism, *Mean*	0.02	[−0.09, 0.13]	0.30
Time × Openness to Experience, *Mean*	−0.05	[−0.20, 0.10]	−0.65
Time × Conscientiousness, *Mean*	0.06	[−0.04, 0.16]	1.15
Time × Agreeableness, *Mean*	−0.04	[−0.21, 0.13]	−0.45
Time × Competency, *Mean*	−0.07	[−0.15, 0.01]	−1.66
Time × Neuroticism, *S.D.*	0.11	[−0.006, 0.23]	1.87
Time × Openness to Experience, *S.D.*	−0.10	[−0.25, 0.04]	−1.38
Time × Conscientiousness, *S.D.*	−0.02	[−0.17, 0.14]	−0.20
Time × Agreeableness, *S.D.*	−0.22	[−0.44, 0.006]	−1.91
Time × Competency, *S.D.*	0.06	[−0.08, 0.21]	0.83
Variance Components			
Level 2 Intercept (Teams)	0.28		
Level 2 Slope (Teams)	0.16		
Intercept-Slope Covariance (Teams)	−0.52		
Level 1 Intercept (Time)	0.43		
Level 1 Slope (Time)	0.06		
Intercept-Slope Covariance (Time)	0.57		
Within-Team Variance	0.44		
*AIC*	1382.87		
*BIC*	1530.96		
−*2LI*	1316.86		
Overall Pseudo-*R^2^_m_*	0.09		

*Note*. All models are random intercept, random slopes models, −2 LL = −2 log-likelihood value. For Model 12, only confidence intervals for fixed effects are reported; confidence intervals for the variance components could not be calculated in *R* due to a non-positive definite in the variance-covariance matrix. Values significant according to *95% CI* are bold.

**Table 7:** Hypotheses and support for initial levels and changes in team psychological safety.

	Support?
Hypothesis on the Effect of Time on PS	
H 1: As time passes in a team project, PS decreases.	Supported
Initial Level Hypotheses	
H 2: Demographic faultline strength is negatively associated with initial levels of PS. H 3a: The higher a team’s mean level in extraversion, the higher the initial levels of PS will be in the team. H 3b: The more diverse team members are in extraversion, the lower initial levels of PS will be in the team. H 3c: The higher a team’s mean level in neuroticism is, the lower initial levels of PS will be.	Supported Not supported Not supported Not supported
Change Level Hypotheses	
H 3d: The more diverse team members are in neuroticism, the more PS will decrease over time. H 3e: The higher a team’s mean level in openness to experience, the more PS within the team will increase over time. H 3f: The more diverse team members are in conscientiousness, the more PS will decrease over time. H 3g: The higher a team’s mean level of agreeableness, the more PS within the team will increase over time. H 3h: The more diverse team members are in agreeableness, the more PS will decrease over time.	Not supported Not supported Not supported Not SupportedSupported
H 4: Team member task-relevant competencies predict changes in PS: The greater a team’s competency, the more PS will decrease over time.	Supported

In Model 1 (Table 2), we found that time significantly predicted changes in PS with PS decreasing over time (*b* = 0.09, *p* = 0.002). Thus, we found support for H1. In Model 2, we predicted initial levels (intercept) of PS with demographic faultline strength and the control variables team diversity regarding gender (Blau index) and semester term (within-team *SD*). Initial levels depended significantly on faultline strength: The stronger the demographic faultline, the lower teams assessed initial levels of PS to be. Thus, we found support for H2.

In Model 3, we predicted initial levels (intercept) of PS by adding deep-level diversity in terms of team member extraversion (H3a regarding mean values and H3b regarding *SD*s), when controlling for effects of demographic faultline strength, gender diversity, and tenure diversity. We found that neither mean levels of team member extraversion nor diversity in team member extraversion predicted initial levels of PS (no support for H3a and H3b).

We repeated this procedure for team member neuroticism separately in Model 4. Contrary to our assumption, it was not the mean value, but diversity in neuroticism (the within-team *SD*) that affected initial levels of PS: The more diverse the team was in neuroticism, the lower teams assessed initial levels of PS to be (no support for H3c). In Model 5, the combined model with all tested predictors is depicted.

In Model 6 (Table 3), we present our baseline model for predicting changes (the slope) in PS including the control variables team size, gender diversity, and tenure diversity. In Model 7 (Table 3), we predicted changes in PS with diversity in team member neuroticism (H3d *SD*s). We, therefore, added the respective interaction (i.e., the product terms) of this predictor with time and found that changes in PS did not depend on diversity in neuroticism (no support for H3d). In the following, we repeated the procedure separately for our deep-level characteristics according to our hypotheses. In Model 8 (Table 4), we predicted changes in PS with team member openness (H3e mean values) and found that changes in PS did not depend on team member openness (no support for H3e). In Model 9 (Table 4), we predicted changes in PS with diversity in team member conscientiousness (H3f *SD*s) and found that changes did not depend on diversity in team member conscientiousness (no support for H3f).

**Figure 1:**
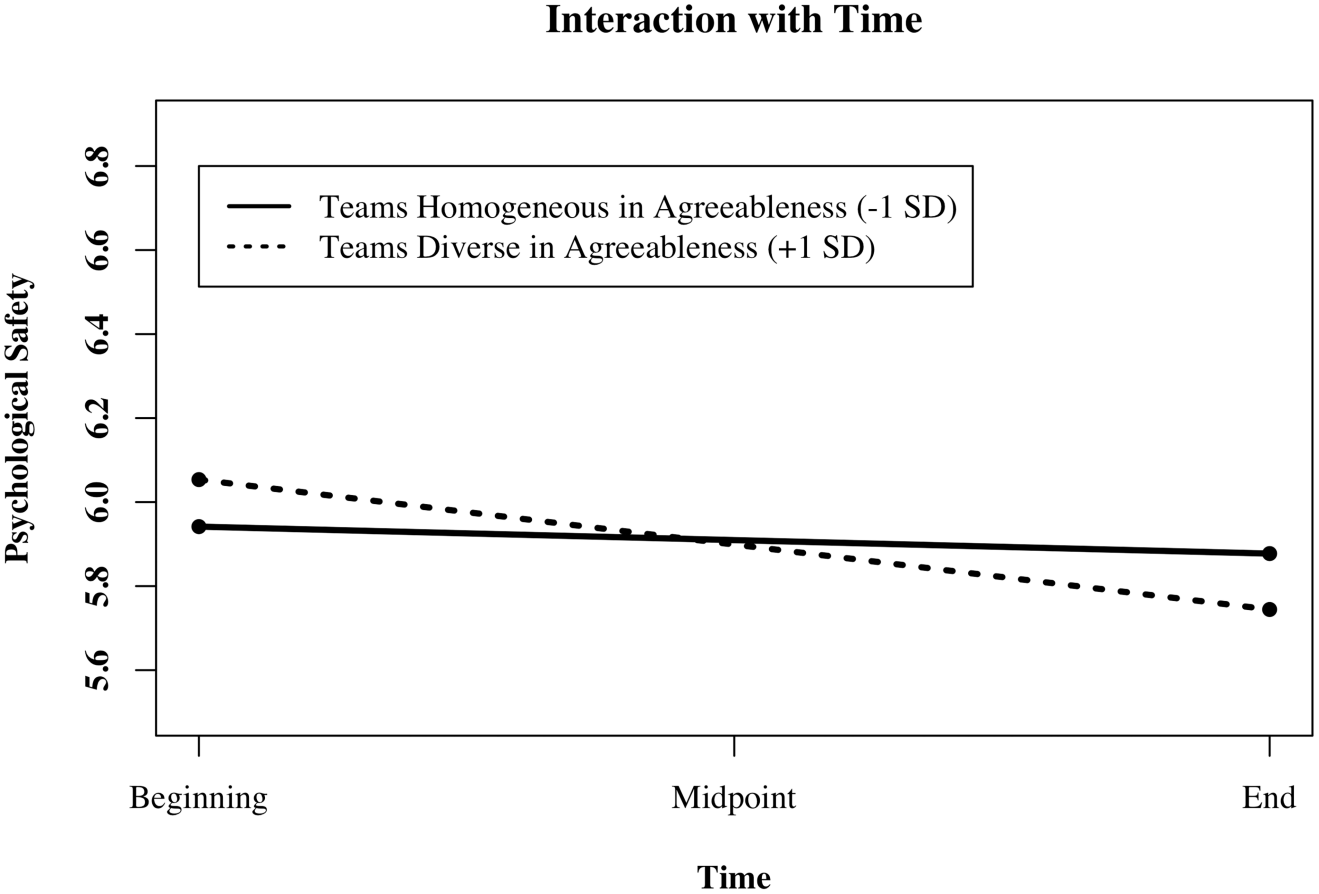
Illustration of the interaction between mean group member diversity in agreeableness (homogeneous vs. diverse) and time predicting changes in psychological safety in research teams (*N* = 699 measurement points in time, nested in 233 members in 61 teams).

In Model 10 (Table 5), we predicted changes in PS with team member agreeableness (H3g mean values) and diversity in agreeableness (H3h *SD*s) and found that changes in PS depended on diversity in team member agreeableness (support for H3h, but no support for H3g). The more diverse team members were in agreeableness, the more did PS decline over time. We plotted this interactive effect of the predictor team member diversity in agreeableness with time and used the web tool provided by Preacher et al. (2006) to test for simple slopes. Figure 1 illustrates how PS changes as a function of team member diversity in agreeableness and time. Psychological safety decreased in teams that were diverse in agreeableness [γ = −0.15 (0.03), *p* < 0.001] and remained stable in teams with homogeneous agreeableness.

**Figure 2:**
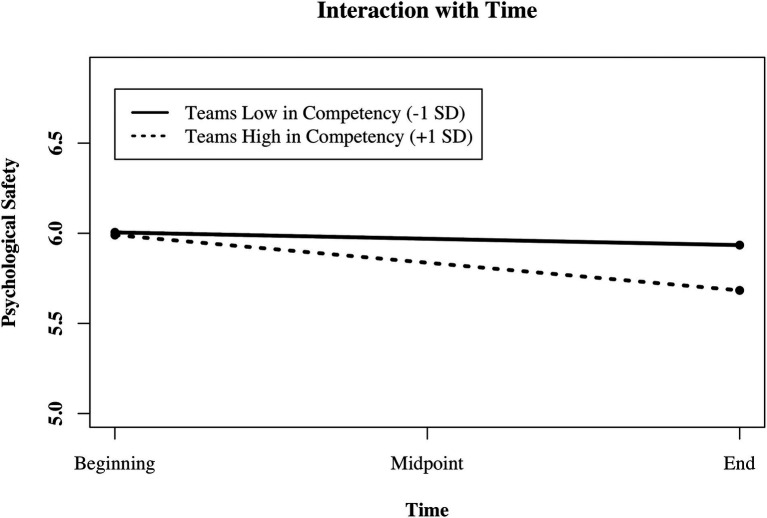
Illustration of the interaction between mean group member competency (low vs. high) and time predicting changes in psychological safety in research teams (*N* = 699 measurement points in time, nested in 233 members in 61 teams).

In Model 11 (Table 5), we predicted changes in PS with team member competencies (H4 mean values) and found that changes in PS depended on team members’ competencies (support for H4). The more competent team members were, the more did PS decline over time. We plotted the interactive effect of the predictor team member competencies with time to test for simple slopes (Preacher et al., 2006). Figure 2 illustrates how PS changes as a function of team member competencies and time. Psychological safety decreased in teams with relatively high member competencies [γ = −0.15 (0.04), *p* = 0.0003] and remained stable in teams with relatively low competencies. Residuals for all reported models were normally distributed so that the requirements for computing mixed models were met. We estimated the overall variance explanation of the models with the pseudo-R^2^ for generalized mixed-effect models (Nakagawa and Schielzeth, 2013).

In Model 12 (Table 6), all control variables and predictors are included simultaneously, for the reader’s information. In summary, we found that PS significantly decreased over time. Initial levels of PS depended on demographic faultline strength and team members’ diversity in neuroticism: The stronger the faultlines were, and the more diverse team members were in their neuroticism, the lower they assessed initial levels of PS to be. Changes in PS depended on team member diversity in agreeableness and on team member competencies. PS decreased more strongly over time when team members were diverse in agreeableness and assessed their task-related competencies to be relatively high.

## Discussion

This study serves to deepen our understanding of the dynamics of PS. It sheds a light on how PS forms and develops over time in the context of member diversity—from the very beginning to the end of project teams. In a sample of self-managed teams, we found that PS decreased over the course of a team project. Initial levels in PS were especially low when teams had strong faultlines and team members were diverse in neuroticism. Changes in PS depended on team member agreeableness and competencies such that PS decreased to a stronger extent when team members varied in their agreeableness and assessed their task-related competencies to be high. We start our discussion by addressing the development of PS found in our study: Most teams started at initially high levels, which significantly decreased over time.

The decrease in PS over time is in line with the descriptive statistics reported by Schulte et al. (2012). There are two explanations for the decline in PS: First, at the beginning of a team project, it is commonly acceptable to allow many questions from team members to form a shared mental model of the task and a transactive memory system about members’ areas of expertise (Gockel and Brauner, 2013). Also, at this early stage, not many problems need to be addressed because they have not occurred or are not obvious yet. However, after this initial period, most team members may think that the task is clear and that first actions should be taken. In this phase, additional questions and feedback could be perceived as rather irritating and may slow down the work. Yet, because the first sessions set standards, PS is very high at the beginning, followed by a consecutive decrease over time. At the finish line, the final critical consideration of one’s teamwork is important for perceived team performance; however, the team may already be too “closed off” to reopen the discussion or allow feedback loops, and this closing phenomenon is reflected in a decrease in PS. Two findings support this consideration: Teams with a high need for closure, which might be triggered by time pressure, are generally less open to critical discussion (Pierro et al., 2003). In addition, the acceptance of novel contributions decreases as time passes in teams facing a deadline (Ford and Sullivan, 2004). Particularly around the temporal midpoint, teams appear to experience a transition between two work orientations that provides a starting point for interventions to increase or stabilize PS (Ford and Sullivan, 2004; Knight, 2015). Our results support a continuous decrease over a nonlinear decrease. The team’s assessment of risk-taking before asking questions or seeking support (and not just task information) decreases from beginning to end across all teams depending on the time passed. Altogether, this suggests that PS as a supportive learning environment has a boundary concerning time or specific temporal milestones—at least in teams with a clear deadline.

The second explanation for the decline in PS refers to a phenomenon found in trust research (Lewicki et al., 2006). It is reasonable that, at their first meeting, team members evaluate their PS based on other criteria than on their experience with risky behavior in the team, because they may not yet have had enough experience with this specific team. For instance, they could have used the teams’ structure (self-managed teams without formal leaders) or other status signals such as gender, age, or floor-gaining behavior for first impressions. Thus, members give a leap of faith and PS to the team, which is to be evaluated later during teamwork (reflected in lower team agreements at midpoint). Perhaps, up until this point, team members shared the anticipated credence of PS. Enthusiasm at the start as a kind of initial ignition and its reduction over time appears not to be unusual for teamwork (see also, e.g., the romance of teams or the honeymoon-hangover effect from research on newcomers’ job satisfaction, Allen and Hecht, 2004; Boswell et al., 2005).

In our study, initial levels of PS depended on demographic faultline strength. Our findings complement previous research in this area. For example, teachers reported lower individual levels of PS in the presence of strong team faultlines compared to weak faultlines (Gerlach and Gockel, 2018). The present study is the first to demonstrate the temporal link between team faultlines and initial levels of PS. This is in line with a discussion by Lau and Murnighan (2005) that strong demographic faultlines have a “direct and potentially pervasive effect” (p. 655) on team processes right from the start. As such, team members are more likely to identify with their subgroup and share sensitive information with close members of their subgroup instead the entire team, which leads to more isolated communication and information sharing and an overall reduced PS (Lau and Murnighan, 2005).

Different from our expectation, team members’ diversity in neuroticism (and not mean values) predicted the formation of PS. The more diverse team members were in neuroticism, the lower teams assessed their PS at the beginning to be. This finding adds to former research that stressed the mean value compared to diversity in neuroticism to be important for early levels of PS (Ostermeier et al., 2020). One explanation for our finding could be that teams diverse in neuroticism scores felt initially uncomfortable in the team, as members perceived others to be different in their way of communication. This might hamper a smooth collaboration and all communication behaviors that are associated with PS right from the start (Barrick et al., 1998). Nevertheless, this relation should be interpreted with caution because we did not hypothesize the effect to occur. It needs future research to test its robustness.

If neuroticism is an important concept for communication behavior, especially if individuals have extreme values, future studies might explore the influence of single members on the creation of PS. For future research, the use of other operationalizations such as taking the minimum or maximum or skewness of a trait into consideration might bring new insights into the possible dynamics of PS (see Bell, 2007; Ostermeier et al., 2020). Still, it is important to note that only neuroticism, and not extraversion, was found to predict the formation of PS. Future research might focus on attitudes that are important for team performance such as team members’ orientation toward teamwork (Ulloa and Adams, 2004).

Changes in PS over time depended on only one personality characteristic, namely team members’ agreeableness. The more team members varied in their agreeableness, the more PS decreased throughout the project. In other words, compared to teams homogeneous in agreeableness, teams diverse in agreeableness started with relatively high PS but decreased more steeply over time. We assume that in these diverse teams, the more agreeable members create a trustful, warm, and tolerant atmosphere, in which the more disagreeable members feel comfortable taking risks and pushing for learning. This behavior could be beneficial for PS at the beginning of the team interaction but lead to more disagreements, conflicts, and a deterioration in PS over time. In teams homogeneous in agreeableness, PS seems rather stable throughout the project. This might create a holding environment for risk-taking behavior. Our study is the first to test the link between team member agreeableness and PS, to our knowledge. The personality characteristic agreeableness has lately received increased attention as an important predictor of team cohesion and team performance, especially if teams work face-to-face (Bradley et al., 2013). Recent research showed that mean levels of agreeableness in teams predicted an increase in team cohesion (Acton et al., 2020). Our results add to these findings by stressing team members’ variation over mean values of agreeableness for the development of PS. A fruitful avenue for future research is to focus on specific behaviors of agreeable and disagreeable team members and their mutual influence over time to tease apart the more positive and negative effects on PS and subsequent learning behavior. In this context, accountability could be a promising element in explaining why also low PS might be beneficial for producing learning behavior (Weiner et al., 2021).

Changes in PS did not depend on team member neuroticism, openness to experience, or conscientiousness in our study. Thus, our findings only partly support the idea that PS unfolds based on team members’ personality characteristics. This adds to Edmondson and Mogelof’s (2005) findings in innovative teams, where openness and neuroticism (at an individual level) predicted perceptions of PS at the midpoint and the end of the collaboration. It is plausible that if teams understand the value of PS for innovation as an index of team performance, other personality facets might have a greater effect on PS depending on their relevance for task performance. Further, our findings are in line with recent results that could not support the hypothesis that mean values in conscientiousness predict the development of PS (Ostermeier et al., 2020).

As a second predictor for changes, we identified team members’ task-related competencies. The higher team members rated their competencies to be at the beginning of the project, the more PS decreased throughout the project. Members of a group with high competencies are more likely to believe that they are right, stick to their positions, and disregard the opinions of others (Innami, 1994). In a previous study, groups that needed to find a consensus to solve the NASA moon landing task showed different communication patterns dependent on the diversity of their task-relevant skills (Innami, 1994). As such, groups with high task-relevant competencies held on to their positions (positional orientation) and did not exchange reasons (reasoning orientation). Time pressure may strengthen this communication behavior as group members fail to understand the importance of explaining task-related issues to each other.

When time pressure increases toward the end of the project, the team has to organize itself with regards to its performance: What needs to be completed by whom and when? These actions require an internal leadership structure and a tacit knowledge of who knows what and has which skills. Personality characteristics such as openness are linked to role development from the beginning (Delice et al., 2019). Others such as extraversion are linked to being perceived as leader-like. The personality of single team members may translate into team emergent states like PS through single behavior or joint sensitivities (LePine et al., 2011) as we found to be true for neuroticism and agreeableness. Dynamics like this can be described in theoretical models as we have suggested above. Nevertheless, there might be critical incidents in situations, task requirements, or other individual unique constellations that lead to experiences made by the team that have a stronger impact on the development of PS, and especially on the development of different PS perceptions in subgroups. Future research regarding team members’ personality characteristics should focus more on the impact of personality and situation settings or consider subgroup dynamics through team members’ perception of subgroups and subsequent differences in team members’ PS.

### Strengths and limitations

We addressed scholarly calls for more holistic research regarding time and changes in teams, more specifically regarding the formation and changes in PS (Roe et al., 2012; Edmondson and Lei, 2014). We refer to theories from diversity research and the model of team faultlines to provide a theoretical frame for how PS forms (Lau and Murnighan, 2005). Another strength of our study is the focus on the team itself, specifically on the effects of deep-level team diversity as antecedents for the development of PS. We help build the nomological net of how personality characteristics at the team level relate to team assessments of PS at different time points of a team project. Further, we add insights to previous research on select personality characteristics at the individual or team level (Edmondson and Mogelof, 2005; Ostermeier et al., 2020). Our study is one of the rare longitudinal team studies that follows teams over an entire project period. Finally, we used sophisticated methods such as multilevel growth curve modeling to predict initial levels and changes in PS based on this longitudinal design to match the dynamic nature of PS.

A few limitations of this study must be pointed out. These teams are characterized by demographic homogeneity (e.g., age, gender, educational background, and experiences in teamwork). A few teams may have been self-formed. Thus, our results should be generalized with caution. The study should be replicated with different kinds of teams in various organizational contexts; thereby controlling for other elements in the team’s structure such as hierarchical differences or team member orientation, for example toward achieving the goal of the task (e.g., Acton et al., 2020). Nevertheless, our study’s setting fulfilled the requirements for self-managed teamwork. As discussed by Lau and Murnighan (2005), we expect demographic faultline strength to have an even greater impact on PS in teams, whose members are more diverse in terms of gender, age, tenure, and educational backgrounds, than the teams in our study. Our findings are only partly in line with our assumptions on the effects of team personality on the dynamics of PS. Many hypothesized effects we did not find. Therefore, we encourage researchers to address this topic in future research, for example event-based, when critical circumstances or incidents such as voice make PS a priority in the team to identify factors in member personality characteristics that predict changes – such as the decrease over time that we found (*cf.* event system theory by Morgeson et al., 2015, applied to employees’ voice in Li and Tangirala, 2021).

### Implications

Our findings allow some preliminary conclusions about how and when to intervene more effectively to support the development of PS. Focusing on team properties and team member characteristics helps to predict a psychologically safe atmosphere for teamwork. We found that demographic faultline strength and some facets of deep-level diversity affected initial levels as well as changes in PS. Our study stresses the importance of states and changeable variables, namely task-relevant competencies, group dynamics of only two traits, neuroticism for initial levels, and agreeableness for changes in PS. The fact that PS decreased over time highlights the importance of time-related team interventions at the beginning, midpoint, and end (*cf.* Hackman and Wageman, 2005).

Therefore, at the beginning of a project, we recommend stressing pro-diversity beliefs in combination with enhanced task motivation to overcome the negative effects of team faultline strength (Meyer and Schermuly, 2012). The goal should be that team members develop positive and realistic expectations toward teamwork and will understand the value of diverse perspectives in teams. Later, during task work, we recommend training communication skills by stressing the importance of explaining one’s view to the team and not holding on to positions without further elaboration (e.g., communication training for teams by Innami, 1994). This is especially advisable for teams of experts with high abilities because our results showed a decrease in PS in such teams over time. Toward the end, a reminder of the previous intervention (showing that diverse opinions are enriching and a strong focus on work is essential to improve output) could help in keeping the team open and not let it close too early to make final changes that could substantially improve the project. Future studies might explore the dynamics of subgroups from the subjective perspective of team members in bigger and more diverse teams, as well as how changes in PS affect team processes such as team performance, and how a decrease might be repaired or rebuilt for single members, or the team as a whole.

### Conclusion

Our study shows that PS changes throughout a team project and how it depends on the team’s faultline strength, diversity in team member neuroticism and agreeableness, and team member competencies. Our study contributes to research on PS by drawing from the theory of subgroups to explain team dynamics in PS and by shifting the focus from the leader (who is often seen as one primary source of influence) to the team as a whole. It introduces a theoretical framework for temporal dynamics in PS and provides empirical evidence on how PS forms and develops over time. It demonstrates the consistently negative effect of demographic faultlines on PS and that only two of the five tested personality characteristics translated to PS on the team level. Finally, our findings stress the importance of time when designing interventions to enhance PS in teams.

## Data availability statement

The raw data supporting the conclusions of this article will be made available by the authors, without undue reservation.

## Ethics statement

Ethical review and approval was not required for the study on human participants in accordance with the local legislation and institutional requirements. The participants provided their written informed consent to participate in this study.

## Author contributions

This article has been an intensive collaboration between the two authors. RG and CG jointly conceptualized the study, developed the hypotheses, and conducted the study at Chemnitz University of Technology. RG ran the analyses. RG and CG developed the manuscript together with RG writing the draft and CG providing feedback. Both authors significantly contributed to the article and approved the submitted version.

## Funding

This research was funded by the Deutsche Forschungsgemeinschaft (DFG, German Research Foundation) project number 491193532 and Technische Universität Chemnitz. Any research material or data can be accessed via email from the RG.

## Conflict of interest

The authors declare that the research was conducted in the absence of any commercial or financial relationships that could be construed as a potential conflict of interest.

## Publisher’s note

All claims expressed in this article are solely those of the authors and do not necessarily represent those of their affiliated organizations, or those of the publisher, the editors and the reviewers. Any product that may be evaluated in this article, or claim that may be made by its manufacturer, is not guaranteed or endorsed by the publisher.
